# Clinical symptoms and epidemiological survey of early-onset severe obesity among children and adolescents

**DOI:** 10.12669/pjms.41.5.10287

**Published:** 2025-05

**Authors:** Miao Wang, Dawei Tian, Jinlin Han, Ning Chen, Humeng Wu

**Affiliations:** 1 Miao Wang Department of Paediatrics, Maternity & Child Care Center of Qinhuangdao, Qinhuangdao 066000, Hebei, China; 2 Dawei Tian Department of Paediatrics, Maternity & Child Care Center of Qinhuangdao, Qinhuangdao 066000, Hebei, China; 3 Jinlin Han Department of Paediatrics, Maternity & Child Care Center of Qinhuangdao, Qinhuangdao 066000, Hebei, China; 4 Ning Chen Department of Children’s Health Care, Maternity & Child Care Center of Qinhuangdao, Qinhuangdao 066000, Hebei, China; 5 Humeng Wu Department of Aural Sursery, Lulong County Hospital, Qinhuangdao 066400, Hebei, China

**Keywords:** Adolescents, Children, Clinical symptoms, Early stage, Epidemiological survey, Severe obesity

## Abstract

**Objective:**

To investigate the clinical symptoms and conduct an epidemiological survey of early-onset severe obesity among children and adolescents in Qinhuangdao City of China from 2022 to 2023.

**Methods:**

This was a retrospective study two-hundred and fifty children and adolescents diagnosed with early-onset severe obesity from August 2022 to August 2023 at Maternity & Child Care Center of Qinhuangdao were selected as subjects; additionally, two-hundred and fifty cases of healthy children and adolescents undergoing routine medical examinations in the same period were selected as the non-obese group in a 1:1 ratio. Logistic regression analysis was employed to identify factors associated with the occurrence of early-onset severe obesity among children and adolescents.

**Results:**

Predominantly, early-onset severe obesity was observed in individuals aged over 14 years, females, those from families with a monthly income per capita of 5000 RMB, and children of obese parents or parents with lower educational levels. The binary logistic regression model identified several significant predictors of severe obesity, including parental obesity, maternal education level (junior high school and above), paternal education level (junior high school and above), non-picky eating habits, eating speed (faster), eating habits, daily outdoor activity duration (>1 hour), and average daily sleep duration (>8 hours) (p<0.05).

**Conclusion:**

Parental obesity, maternal education level (junior high school and above), paternal education level (junior high school and above), eating habits, daily outdoor activity duration (>1 hour), and average daily sleep duration (>8 hours) may be significant factors influencing the occurrence of severe obesity in children and adolescents.

## INTRODUCTION

The prevalence of obesity among children and adolescents is escalating globally, posing a substantial challenge to public health. Particularly, early-onset severe obesity demands increased focus due to its implications on both physical health and psychosocial well-being.^1^-^3^ In Qinhuangdao, a densely populated and economically dynamic city, a rising trend in the incidence of early-onset severe obesity among children and adolescents has been observed.^4^,^5^

Comprehending the clinical symptoms of early-onset severe obesity among children and adolescents facilitates the prompt recognition and intervention of this condition, thus alleviating its detrimental impacts on the physical and mental health of these individuals. Concurrently, conducting epidemiological surveys enables the acquisition of data on the incidence, trends, and contributory factors of early-onset severe obesity among children and adolescents in the region, providing a robust scientific foundation for devising effective prevention and control strategies.^6^,^7^ This study aimed to investigate the clinical manifestations and contributory factors of early-onset severe obesity in this demographic within Qinhuangdao over the years 2022-2023.

## METHODS

This was a retrospective study total of 250 children and adolescents diagnosed with early-onset severe obesity from August 2022 to August 2023 at Maternity & Child Care Center of Qinhuangdao were selected as subjects. Additionally, two-hundred and fifty cases of healthy children and adolescents undergoing routine medical examinations in the same period were selected as the non-obese group in a 1:1 ratio. All participant data included the medical data from the Maternity & Child Care Center of Qinhuangdao information and management system, collected their various information of them.

### Ethical Approval:

The study was approved by the Institutional Ethics Committee of Maternity & Child Care Center of Qinhuangdao (No.: QHDFY-2023041909; Date: April 19, 2023), and written informed consent was obtained from all parents/guardians of the participants.

### Inclusion criteria:

- Meeting the diagnostic criteria for early-onset severe obesity (BMI > 25 kg/m² for children under two years, > 30 kg/m² for children aged 2-6 years, 35 kg/m² for children aged 6-14 years, and > 40 kg/m² for children older than 14 years).
- Aged between 1~18 years.
- Exhibiting behaviors of binge eating and food-seeking.
- Acquisition of written informed consent from parents/guardians and patients aged 13 years and above, with complete relevant data.

### Exclusion criteria:

- Substantial organ dysfunction.
- Diagnosed genetic syndromes coexisting with obesity, medications known to affect weight gain (e.g., corticosteroids, valproate sodium, risperidone), Cushing’s syndrome, and other causes of obesity.
- Primary psychiatric diseases, consciousness disorders, and cognitive impairments.

A survey questionnaire, refined from the 2013 version for simple obesity in children under six years of age, was employed.^8^ The questionnaire included variables such as age, gender, household monthly income per capita, left-behind children, only child status, parental obesity, maternal education level, paternal education level, reconstituted families, picky eating habits, eating speed, meat-vegetable ratio, breakfast consumption, preference for meat, western fast food, snacks & beverages, binge eating, parental awareness of obesity prevention, parental understanding of the adverse consequences of obesity, daily outdoor activity duration, average daily sleep duration, and frequency of dining out. The survey was conducted by specially trained personnel, and the questionnaire was filled out by the parents of the participating children.

### Statistical Analysis:

All data were statistically analyzed using the SPSS 22.0 software (SPSS Inc., Chicago, IL, USA). The confidence interval was 95%. Enumeration data were expressed as (n, %), and the chi-squared test or Fisher’s exact test was used for statistical analysis. Measurement data following a normal distribution were presented as *χ̅±S* and analyzed using the student’s t-test. P< 0.05 was considered a statistically significant difference.

## RESULTS

Predominantly, early-onset severe obesity was observed in individuals aged over 14 years, females, those from families with a monthly income per capita of 5000 RMB, and children of obese parents or parents with lower educational levels (Table-I).

**Table-I:** Analysis of clinical characteristics of 250 pediatric patients with early-onset severe obesity.

Index	Classification	Severe obesity (250)	Composition ratio (%)
Age	Under 2 years old	32	12.80
	2-6 years old	69	27.60
	6-14 years old	70	28.00
	>14 years old	79	31.60
Gender	Male	123	49.20
	Female	127	50.80
Household monthly income per capita	<3000 RMB	22	8.80
	3000-5000 RMB	155	62.00
	>5000 RMB	73	29.20
Parental obesity	Yes	22	8.80
	No	228	91.20
Maternal education level	Elementary school and above	60	24.00
	Junior high school/high school/technical secondary school	155	62.00
	University and above	35	14.00
Paternal education level	Elementary school and above	46	18.40
	Junior high school/high school/technical secondary school	162	64.80
	University and above	42	16.80

Univariate analysis revealed no statistically significant differences between the two groups in terms of age, gender, household monthly income per capita, left-behind children, only child status, and blended family (*p>*0.05); however, significant differences were observed in parental obesity, maternal education level, and paternal education level (*p<*0.05) (Table-II).

**Table-II:** Univariate analysis of general data of the two groups [*n* (%)].

Index	Classification	Non-obese group (250)	Severe obesity (250)	χ^2^	P
Age	Under 2 years old	28 (11.20)	32 (12.80)	0.220	0.639
	2-6 years old	71 (28.40)	69 (27.60)		
	6-14 years old	68 (27.20)	70 (28.00)		
	>14 years old	83 (33.20)	79 (31.60)		
Gender	Male	110 (44.00)	123 (49.20)	1.358	0.244
	Female	140 (56.00)	127 (50.80)		
Household monthly income per capita	<3000 RMB	18 (7.20)	22 (8.80)	2.280	0.131
	3000-5000 RMB	142 (56.8)	155 (62.00)		
	>5000 RMB	90 (36.00)	73 (29.20)		
Left-behind children	Yes	65 (26.00)	69 (27.60)	0.163	0.686
	No	185 (74.00)	181 (72.40)		
Only child status	Yes	42 (16.80)	37 (14.80)	0.376	0.540
	No	208 (83.20)	213 (85.20)		
Parental obesity	Yes	6 (2.40)	22 (8.80)	9.685	0.002
	No	244 (97.60)	228 (91.20)		
Maternal education level	Elementary school and above	24 (9.60)	60 (24.00)	28.44	<0.001
	Junior high school/high school/technical secondary school	151 (60.40)	155 (62.00)		
	University and above	75 (30.00)	35 (14.00)		
Paternal education level	Elementary school and above	20 (8.00)	46 (18.40)	13.017	<0.001
	Junior high school/high school/technical secondary school	168 (67.20)	162 (64.80)		
	University and above	62 (24.80)	42 (16.80)		
Blended family	Yes	175 (70.00)	157 (62.80)	2.904	0.088
	No	75 (30.00)	93 (37.20)		

Univariate analysis showed that there were statistically significant differences between the two groups in terms of picky eating habits, eating speed, meat-vegetable ratio, breakfast consumption, preference for meat, western fast food, snacks & beverages, binge eating, parental awareness of obesity prevention, parental understanding of the adverse consequences of obesity, frequency of dining out, daily outdoor activity duration, and average daily sleep duration (*p<*0.05) (Table-III).

**Table-III:** Univariate analysis of life habits of the two groups [*n* (%)].

Index	Classification	Non-obese group (250)	Severe obesity (250)	χ^2^	P
Picky eating habits	No	56 (22.40)	97 (38.80)	15.831	<0.001
	Yes	194 (77.60)	153 (61.20)		
Eating speed	Faster	28 (11.20)	57 (22.80)	15.399	<0.001
	Normal	102 (40.8)	109 (43.60)		
	Slower	120 (48.00)	84 (33.60)		
Meat-vegetable ratio	Preference for vegetables	45 (18.00)	23 (9.20)	11.007	0.001
	Preference for meat	75 (30.00)	104 (41.60)		
	Balanced meat-to-vegetable	130 (52.00)	123 (49.20)		
Breakfast consumption	Daily	194 (77.60)	160 (64.00)	11.183	0.001
	Not daily	56 (22.40)	90 (36.00)		
Preference for meat	Yes	144 (57.60)	187 (74.80)	16.527	<0.001
	No	106 (42.40)	63 (25.20)		
Preference for western fast food	Yes	130 (52.00)	180 (72.00)	21.222	<0.001
	No	120 (48.00)	70 (28.00)		
Preference for snacks & beverages	Yes	164 (65.6)	195 (78.00)	9.492	0.002
	No	86 (34.40)	55 (22.00)		
Binge eating	Yes	82 (32.80)	106 (42.40)	4.91	0.027
	No	168 (67.20)	144 (57.60)		
Parental awareness of obesity prevention	Yes	117 (46.80)	85 (34.00)	8.506	0.004
	No	133 (53.20)	165 (66.00)		
Parental understanding of the adverse consequences of obesity	Yes	104 (41.60)	77 (30.80)	6.313	0.012
	No	146 (58.40)	173 (69.20)		
Frequency of dining out	Frequently	22 (8.80)	59 (23.60)	20.169	<0.001
	Occasionally	228 (91.20)	191 (76.40)		
Daily outdoor activity duration	<1h	11 (4.40)	32 (12.80)	19.583	<0.001
	1-2h	91 (36.40)	115 (46.00)		
	>2h	148 (59.20)	103 (41.20)		
Average daily sleep duration	≤8h	54 (21.60)	88 (35.20)	11.37	0.001
	>8h	196 (78.40)	162 (64.80)		

Logistic regression analysis was conducted. The results indicated that parental obesity, maternal education level (junior high school and above), paternal education level (junior high school and above), non-picky eating habits, eating speed (faster), meat-vegetable ratio (balanced meat-to-vegetable)/meat-vegetable ratio (preference for meat), breakfast consumption (not daily), preference for meat, western fast food, snacks & beverages, parental awareness of obesity prevention, frequency of dining out (frequent), daily outdoor activity duration (>1 hour), and average daily sleep duration (>8 hours) were significant factors influencing the occurrence of severe obesity (*p<*0.05) (Table-IV and V).

**Table-IV:** Variable assignment.

Assignment	
Severe obesity	1=No, 2=Yes
Parental obesity	1=Yes, 2=No
Maternal education level	1=Elementary school and above, 2=Junior high school/high school/technical secondary school, 3=University and above
Paternal education level	1=Elementary school and above, 2=Junior high school/high school/technical secondary school, 3=University and above
Picky eating habits	1=No, 2=Yes
Eating speed	1=Faster, 2=Normal, 3=Slower
Meat-to-vegetable ratio	1=Preference for vegetables, 2=Preference for meat, 3=Balanced meat-to-vegetable
Breakfast consumption	1=Daily, 2=Not daily
Preference for meat	1=Yes, 2=No
Preference for western fast food	1=Yes, 2=No
Preference for snacks & beverages	1=Yes, 2=No
Binge eating	1=Yes, 2=No
parental awareness of obesity prevention	1=Yes, 2=No
Parental understanding of the adverse consequences of obesity	1=Yes, 2=No
Daily outdoor activity duration	1=<1h, 2=1-2h, 3=>2h
Average daily sleep duration	1=≤8h, 2=>8h
Frequency of dining out	1=Frequently, 2=Occasionally

**Table-V:** Binary Logistic regression influencing the occurrence of severe obesity.

Variable	B	S.E.	Wald	P	OR	95% C.I.	
						Lower limit	Upper limit
Parental obesity	1.309	0.539	5.892	0.015	3.702	1.287	10.652
Maternal education level			15.278	<0.001			
Maternal education level (Junior high school/high school/technical secondary school)	-0.967	0.312	9.619	0.002	0.380	0.206	0.701
Maternal education level (University and above)	-1.415	0.367	14.889	<0.001	0.243	0.118	0.499
Paternal education level			5.489	0.064			
Paternal education level (Junior high school/high school/technical secondary school)	-0.719	0.343	4.399	0.036	0.487	0.249	0.954
Paternal education level (University and above)	-0.899	0.399	5.074	0.024	0.407	0.186	0.89
Non-picky eating habits	0.759	0.238	10.197	0.001	2.136	1.341	3.403
Eating speed			8.812	0.012			
Eating speed (faster)	0.934	0.317	8.683	0.003	2.545	1.367	4.738
Eating speed (normal)	0.352	0.240	2.151	0.142	1.422	0.888	2.275
Meat-to-vegetable ratio			10.237	0.006			
Meat-to-vegetable ratio (balanced meat-to-vegetable)	1.107	0.373	8.820	0.003	3.026	1.457	6.282
Meat-to-vegetable ratio (preference for meat)	1.119	0.364	9.432	0.002	3.062	1.499	6.254
Breakfast consumption (not daily)	0.507	0.237	4.573	0.032	1.661	1.043	2.644
Preference for meat	0.579	0.236	5.992	0.014	1.783	1.122	2.834
Preference for western fast food	0.805	0.225	12.775	<0.001	2.237	1.439	3.479
Preference for snacks & beverages	0.724	0.241	9.026	0.003	2.062	1.286	3.306
Binge eating	0.348	0.224	2.403	0.121	1.416	0.912	2.198
Parental awareness of obesity prevention	-0.436	0.221	3.886	0.049	0.647	0.419	0.997
Parental understanding of the adverse consequences of obesity	-0.437	0.225	3.771	0.052	0.646	0.416	1.004
Frequency of dining out (frequent)	1.050	0.276	14.464	<0.001	2.857	1.663	4.907
Daily outdoor activity duration			14.341	0.001			
Daily outdoor activity duration (1-2h)	-0.844	0.387	4.755	0.029	0.430	0.201	0.918
Daily outdoor activity duration (>2h)	-1.308	0.382	11.726	0.001	0.270	0.128	0.572
Average daily sleep duration (>8h)	-0.611	0.211	8.407	0.004	0.543	0.359	0.820
Constant	1.293	0.394	10.775	0.001	3.642		

## DISCUSSION

The survey findings reveal that among the 250 cases of early-onset severe obesity among children and adolescents, a significant proportion occurred in individuals aged over 14 years, females, those from families with a monthly income per capita of 5000 RMB, and children of obese parents or parents with lower educational level, aligning with the findings of Altschul DM.^9^ The possible reasons include:

### Age:

In the cohort, the majority of patients are older than 14 years. This trend may stem from age-related shifts in dietary preferences and lifestyle choices, such as a preference for high-calorie, high-fat diets and reduced physical activity, culminating in weight gain.^10^

### Gender:

Females represent the majority of these cases. This prevalence may be linked to hormonal fluctuations during female pubertal development, which can precipitate weight gain. Additionally, heightened concerns regarding body image and appearance among females may result in eating disorders and excessive dieting.^11^

### Family economic status:

These patients come from families with an average monthly income of 5000 RMB. This may indicate that families with superior economic conditions might more readily afford high-calorie, high-fat foods, or exhibit less healthy eating habits. Furthermore, a higher family income could imply that parents have limited time and energy to devote to managing their children’s dietary and exercise habits.^12^

### Parental obesity:

A heightened incidence of obesity is observed among parents. This trend could be attributed to hereditary factors, as obesity often has familial ties.^13^,^14^ Moreover, the lifestyle and dietary habits of parents can also impact their offspring.

### Parental education level:

The parents of these patients typically possess lower educational qualifications. This condition may correlate with a deficiency in health education knowledge and awareness among parents, rendering it challenging for those with limited educational levels to offer scientifically sound and healthy dietary and exercise advice to their children. Collectively, these factors underscore that the occurrence of early-onset severe obesity in children and adolescents is a multifaceted process involving the interaction of multiple factors.

Obesity is a multifaceted health challenge shaped by a variety of determinants. Univariate analysis in this study revealed statistically significant differences between the two groups in terms of picky eating habits, eating speed, meat-to-vegetable ratio, breakfast consumption, preference for meat, western fast food, snacks & beverages, binge eating, parental awareness of obesity prevention, parental understanding of the adverse consequences of obesity, frequency of dining out, daily outdoor activity duration, and average daily sleep duration, indicating that these factors may impact early-onset severe obesity in children and adolescents.^15^-^18^

Subsequent analysis employing a binary Logistic regression model in this study showed that parental obesity, maternal education level (junior high school and above), paternal education level (junior high school and above), non-picky eating habits, faster eating speed, balanced meat-to-vegetable ratio/preference for meat, irregular breakfast consumption, preference for meat, western fast food, snacks & beverages, parental awareness of obesity prevention, frequency of dining out (frequent), daily outdoor activity duration (>1 hour), and average daily sleep duration (>8 hours) were significant factors influencing the occurrence of severe obesity in children and adolescents, similar to the findings of Lister NB.^19^

Therefore, children who average more sleep per day have a lower risk of severe obesity. Conversely, insufficient sleep may lead to poor daytime alertness and reduced activity in children, thereby increasing the risk of obesity. Lastly, frequency of dining out (frequent): Regularly eating out can heighten the risk of ingesting high-calorie, high-fat foods, resulting in excessive energy intake and obesity.^20^ Concurrently, society should also enhance the dissemination of health education, raise public awareness of the dangers of obesity, and collectively foster a healthy living environment.

### Limitations:

It includes a small samples size and no follow-up was conducted. In view of this, more samples and follow-up time should be included in future studies to further validate the findings of this study.

## CONCLUSIONS

Parental obesity, maternal education level (junior high school and above), paternal education level (junior high school and above), non-picky eating habits, faster eating speed, balanced meat-to-vegetable ratio/preference for meat, irregular breakfast consumption, preference for meat, western fast food, snacks & beverages, parental awareness of obesity prevention, frequency of dining out (frequent), daily outdoor activity duration (>1 hour), and average daily sleep duration (>8 hours) may be significant factors influencing the occurrence of severe obesity in children and adolescents.

### Authors’ Contributions:

**MW** and **DT:** Carried out the studies, participated in collecting data, and drafted the manuscript, and are responsible and accountable for the accuracy or integrity of the work.

**JH, NC** and **HW:** Performed the statistical analysis and participated in its design. Critical review.

All authors have read and approved the final manuscript.
